# Collision Volume and Contact Exposure Profile in Elite Women’s Rugby Union: Differences Compared with Men

**DOI:** 10.3390/sports14050210

**Published:** 2026-05-19

**Authors:** Diego Hernán Villarejo-García, Carlos Navarro-Martínez, José Pino-Ortega

**Affiliations:** 1 BioVetMed & SportSci Research Group, Faculty of Sport Sciences, University of Murcia, 30720 San Javier, Spain; dvillarejo@um.es (D.H.V.-G.); pepepinoortega@gmail.com (J.P.-O.); 2 Education, Diversity and Quality Research Group, Faculty of Education, University of Murcia, Campus de Espinardo, 30100 Murcia, Spain

**Keywords:** injury prevention, match analysis, gender differences, performance analysis, team sports, six nations

## Abstract

Elite women’s rugby has often been analysed using the male performance model as a reference, despite evidence that women’s rugby presents distinct game demands and potentially different risk profiles. This study aimed to compare the frequency of key contact-related events between elite men’s and women’s rugby. An observational, retrospective, comparative cohort study was conducted using official performance data from 135 international matches from the men’s and women’s Six Nations Championships. Variables were grouped into three categories: Open-Play, Static Phases, and Discipline. Independent samples *t*-tests, Mann–Whitney U tests, and Linear Discriminant Analysis were used to identify sex-based differences. The results showed that men presented a higher frequency of rucks lasting more than 6 s (*p* < 0.001), whereas no significant differences were found in total tackles (*p* = 0.378) or total rucks (*p* = 0.634). In Static Phases, women’s teams recorded significantly more scrums (*p* < 0.001). In Discipline, women conceded fewer free kicks (*p* = 0.003) but received more red cards (*p* = 0.020). In conclusion, elite women’s rugby shares some open-play characteristics with the men’s game but differs in scrum frequency and disciplinary profile, supporting the existence of a distinct contact and risk exposure profile that should be considered when designing training and prevention strategies.

## 1. Introduction

Performance analysis in elite sport has traditionally relied on the male game model as the primary reference framework [1], favouring data derived from male athletes as “gold standards” for the evaluation and preparation of female athletes [2]. In rugby union, recent research has shown that the application of male-derived data has led to an underestimation of intensity in women’s rugby [3]. From this comparative perspective, women’s rugby has often been interpreted as a lower-intensity game. However, although clear anthropometric and physiological differences exist between sexes [4], these differences should not be interpreted as reflecting lower game intensity, but rather as indicative of distinct game models and performance demands [3].

The body of literature comparing elite male and female rugby populations points to the existence of tactical, physical, and injury profile divergences [5]. Studies such as Hughes et al. [6] reported that, in international competitions, men’s teams prioritise territorial occupation, whereas women’s rugby is characterised by greater ball possession and continuity of play. These findings align with those of Scott et al. [7], who identified Open-Play variables as robust predictors of performance in women’s rugby using multivariate models. In contrast, longitudinal analyses in professional men’s rugby, such as that by Kvasnytsya et al. [8] suggest that success in this population remains strongly associated with territorial indicators and effectiveness in static phases. This distinction may also be linked to differences in physical demands. Accordingly, Nolan et al. [9], analysing the Women’s Six Nations Championship, reported a collision frequency (0.46 collisions·min^−1^) exceeding the ranges previously described in studies of elite men’s rugby (0.18–0.44 collisions·min^−1^).

Differences in contact and collision events are clinically relevant, as collisions represent the primary mechanism of injury in elite rugby union [10,11]. Although men’s rugby is often associated with a higher absolute incidence of impact-related trauma [12], epidemiological studies have indicated that female players may present higher relative risks for specific injuries per hour of exposure, such as concussion and anterior cruciate ligament (ACL) injuries [11,13]. Specifically, evidence suggests that the incidence of ACL injuries in women may be up to 5.3 times higher than in men [14], resulting in a greater burden of time-loss injuries [12]. Furthermore, a significant annual increase in injury rates has been observed in recent women’s competitions, in contrast to the stability reported in male cohorts [14]. Regarding concussion, Allan et al. [15] emphasised the need to monitor impact exposure, as it is directly proportional to concussion risk.

In this context, it can be hypothesised that the interaction between sport-specific game demands—particularly exposure during open play—and the biological characteristics of female athletes may generate a distinct risk profile that requires dedicated analysis, rather than direct extrapolation from male-based models [16].

Despite these indications, there remains a need for studies that quantify and compare physical contact exposure profiles in both populations. Therefore, the aim of the present study was to characterize and compare the collision demands and the exposure profile of potentially injurious game actions in elite men’s and women’s rugby union. It was hypothesized that elite women’s rugby would exhibit a distinct structural profile compared to the men’s game. The findings of this work aim to provide objective information that can be used to optimize physical preparation strategies, injury prevention, and the design of recovery and rehabilitation protocols for return to play.

## 2. Materials and Methods

### 2.1. Study Design

This study followed an observational, retrospective, and cross-sectional design, adhering to the STROBE guidelines [17]. The completed STROBE and SORT checklists are provided as Supplementary Materials (Tables S1 and S2).

### 2.2. Participants and Sample

The sample consisted of 270 team-game observations derived from 135 matches involving the Men’s (*n* = 63) and Women’s (*n* = 72) national teams (England, France, Ireland, Italy, Scotland, and Wales) participating in the 2021–2025 editions of the Six Nations Championship. The inclusion criteria were: (i) official matches of the tournament and (ii) availability of complete technical-tactical performance data through the official provider. A team-game observation was defined as the aggregate data for one team in a single match; thus, each match provided two observations. This longitudinal approach allowed for a comprehensive analysis of game profiles across five full competitive cycles for both sexes.

### 2.3. Data Collection

Match performance data were collected using the Hawk-Eye system [18,19]. This system employs player tracking based on triangulation via calibrated cameras [20,21], and serves as the official data source for the Six Nations Championship (https://www.sixnationsrugby.com/en, accessed on 30 April 2025). To assess the reliability of the collected metrics, a concurrent manual verification was conducted on a subsample of 30 matches (15 male and 15 female, representing 22% of the total sample). Manual coding was performed by an expert analyst with over 20 years of experience in rugby union performance analysis. The variables selected for verification included the primary study indicators: Total Tackles, Total Rucks, Total Scrums, and Total Mauls. Inter-method reliability (Hawk-Eye vs. Manual) was assessed using the Intraclass Correlation Coefficient (ICC, model 2,1 for absolute agreement) [22]. Results indicated good-to-excellent consistency for the evaluated contact metrics, with ICC values ranging between 0.78 and 0.86, confirming the validity of the automated dataset for the comparative analysis.

### 2.4. Variables

Performance variables were categorized into three domains: Open-Play, Static Phases, and Discipline. To ensure terminological clarity, in this study Volume is defined as the absolute frequency of technical events (e.g., total number of tackles); Intensity refers to the speed or tempo of actions (e.g., ruck speed); and Load represents the mechanical or physiological demand imposed on the player by the combination of volume and intensity in contact situations (e.g., axial loading in scrums or post-match neuromuscular load). Operational definitions are detailed in Table 1.

### 2.5. Ethical Considerations

This study was conducted in accordance with the principles of the Declaration of Helsinki [23]. Since the research relied exclusively on official and publicly available performance data from elite competitions, individual informed consent from players or teams was not required [24]. 

### 2.6. Statistical Analysis

Data analysis was performed using Python (Python 3.11; pandas 2.2.3; scipy 1.15.2; scikit-learn 1.6.1). Descriptive data are presented as mean (standard deviation, SD). Normality was assessed using the Shapiro-Wilk test. Comparisons between sexes were performed using Independent Samples *t*-tests (for normally distributed variables) or Mann-Whitney U tests (for non-normally distributed variables). The results indicated that only Lineouts per match (W = 0.993, *p* = 0.771) and 3–6 s Rucks (W = 0.985, *p* = 0.129) followed a normal distribution. Conversely, the vast majority of critical variables presented non-normal distributions (*p* < 0.05), including Total Rucks (W = 0.916, *p* < 0.001), Total Tackles (W = 0.942, *p* < 0.001), Scrums per match (W = 0.884, *p* < 0.001), long-duration Rucks (>6 s) (W = 0.854, *p* < 0.001), and Penalties Conceded (W = 0.965, *p* = 0.001).

Effect Sizes (ES) were reported as Cohen’s d or rank-biserial correlation (r_rb_). Effect sizes were interpreted according to Cohen’s guidelines [25]. For parametric comparisons, Cohen’s d was categorized as small (0.20–0.50), medium (0.50–0.80), and large (>0.80). For non-parametric comparisons, the rank-biserial correlation was interpreted as small (0.10–0.30), medium (0.30–0.50), and large (>0.50).

Furthermore, a post-hoc power analysis for the sample size (*n* = 135 matches) revealed that for a medium effect size (d = 0.5) and a significance level of α = 0.05, the statistical power (1− β) was greater than 0.85, supporting the robustness of the sample to detect significant differences.

For the multivariate analysis, Linear Discriminant Analysis (LDA) was employed to identify the combination of variables that best characterized each sex. Although LDA assumes multivariate normality, it has been demonstrated to be robust to violations of this assumption in large sample sizes (*n* = 135), especially when interpretation is based on structural coefficients (r > 0.30) rather than standardized weights. These structural coefficients were used to identify the variables with the greatest discriminant power while mitigating the impact of potential non-normality. Statistical significance was set at *p* < 0.05 for all analyses.

## 3. Results

### 3.1. Descriptive and Univariate Analysis

Table 2 displays the descriptive data and the results of the inferential analysis comparing male and female match demands.

**Figure 1:**
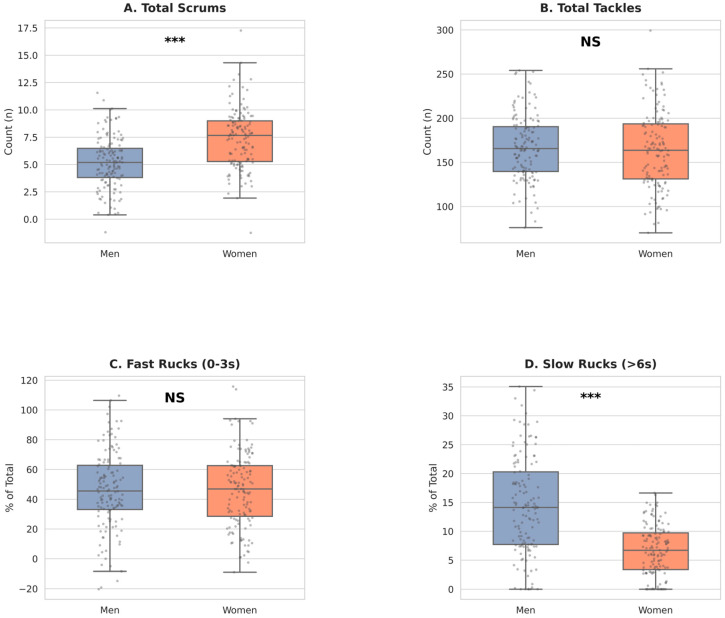
Comparison of: (**A**) Total Scrums; (**B**) Total Tackles; (**C**) Fast Rucks (0–3 s); and (**D**) Slow Rucks (>6 s) between sexes. *** significant difference at *p* < 0.001; NS, not significant.

Figure 1 illustrates the data distributions and the statistical contrast between male and female match demands.

### 3.2. Multivariate Analysis

**Figure 2:**
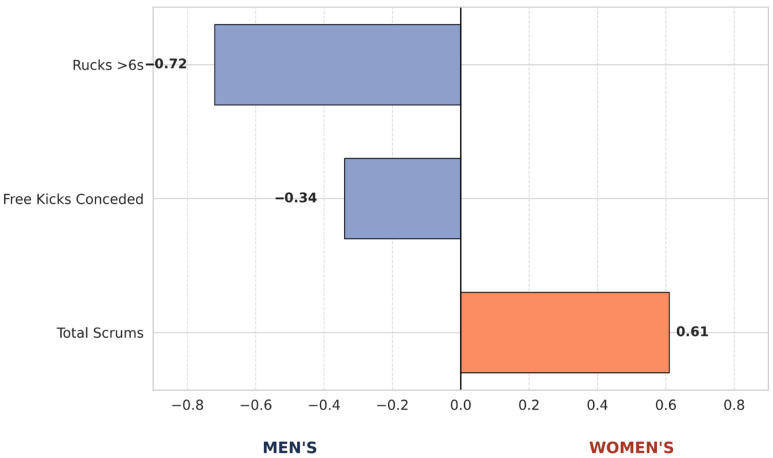
Discriminant Variables of the Match Profile by Sex. Values represent the correlation coefficient (r) with the canonical discriminant function.

The LDA model correctly classified match sex with an accuracy of 71%. The structure matrix (Figure 2) revealed that the frequency of Slow Rucks (r = −0.72) and Total Scrums (r = 0.61) were the primary determinants of group separation. A lower prevalence of slow rucks and a higher density of scrums defined the female profile. In contrast, Total Tackles and Total Rucks exhibited negligible discriminatory power (r < 0.15).

Figure 2 displays the factorial loading of the variables with the greatest discriminatory power between the male and female games. This chart exclusively isolates indicators exceeding the predictive relevance threshold (r > 0.30), visualizing the direction and magnitude of each variable’s association with the respective sex profile.

## 4. Discussion

The primary aim of the present study was to characterise and compare exposure profiles to potentially injurious actions in elite rugby union, in order to determine whether the women’s game model imposes a contact volume different from that of the men’s model. Overall, the results indicate equivalence between sexes in the Open-Play variables, alongside clear differences in the Static Phases and Discipline categories.

More specifically, with regard to the Open-Play category, our findings indicate a marked parity between sexes, challenging the traditional notion that men’s rugby imposes a substantially higher defensive contact volume. The absence of statistically significant differences in the absolute volume of Total Tackles (Attempts) (163.73 vs. 168.26; *p* = 0.378) and Tackles Made (142.29 vs. 146.76; *p* = 0.328) suggests that elite women’s rugby has reached a frequency of collision events comparable to that of the men’s game. This quantitative parity provides a concrete basis for interpreting injury risk; given that female players typically present different anthropometric characteristics and lower muscle mass, the exposure to similar collision volumes implies a proportionally greater relative mechanical demand in women (e.g., contact forces expressed as N × kg^−1^). This interaction is critical for understanding the higher relative risk of concussions reported in female cohorts. These finding nuances previous observations by Nolan et al. [9], who reported higher relative collision frequencies in women. Such parity in exposure volume is clinically relevant, as recent studies in elite rugby have demonstrated that the incidence of head acceleration events is proportional to contact frequency and associated concussion risk [10,12,14,15].

Another relevant finding emerged from the variables describing ruck play. Although the total number of rucks was comparable between cohorts (91.95 vs. 90.53; *p* = 0.634), a significant difference was identified in ruck duration. Men’s competition exhibited a proportion of rucks lasting longer than 6 s that was approximately double that observed in women (12.38% vs. 6.11%; *p* < 0.001), whereas women’s ruck play was characterised by greater fluency, maintaining similar proportions of short rucks (0–3 s; 46%) while avoiding prolonged ruck phases. This difference presents an effect size close to moderate (d = −0.46), indicating a substantial structural disparity in game continuity between sexes. This dynamic may reflect greater exposure of male players to prolonged ball contests at the ruck, potentially translating into an increased risk of contact-related injuries in this formation. In contrast, women’s ruck play, aligned with the continuity-based style described by Hughes et al. [6], may expose players to a higher risk of overuse-related injuries. Nevertheless, these interpretations should be confirmed through direct observation of ruck actions, including pre- and post-ruck phases, and through the integration of instruments capable of quantifying action intensity (e.g., accelerometers and GPS).

In contrast to the patterns observed in open play, substantial differences emerged in the Static Phases category. Women’s rugby may impose a significantly greater load during scrum phases, with an average of 7.21 scrums per match compared with 5.38 in men’s competition (*p* < 0.001). Similarly, the higher frequency of scrums in women shows a moderate effect (d = 0.43), reinforcing the idea that static loading is a distinctive and relevant component of the female game. This profile suggests that female forwards are exposed to a higher axial and static loading component than would be expected based on the male game model. The elevated frequency of scrums may be interpreted as a consequence of handling errors; however, this finding contrasts with traditional perspectives derived from men’s rugby, such as that reported by Kvasnytsya et al. [8], which associate static-phase dominance with male performance success. The present results indicate that contemporary women’s rugby is characterised by a higher frequency of scrum formations. Accordingly, the implementation of specific physical preparation programmes targeting players exposed to the demands of scrummaging is warranted. In practical terms, these programmes may particularly benefit from emphasising cervical spine strength, isometric neck endurance, and trunk stability in female forwards, in order to better tolerate repeated axial loading [26,27]. Strength and conditioning coaches and technical staff should also account for the potentially greater scrum-related intensity experienced by women compared with men by managing in-game exposure and ensuring differentiated post-match recovery strategies.

Finally, the analysis of the Discipline category revealed a complex pattern. Despite the higher frequency of scrums, women’s rugby exhibited significantly lower rates of Free Kicks conceded (0.40 vs. 0.87; *p* = 0.003. While free kicks can be awarded for various infringements across different game phases, they frequently stem from technical errors during set-pieces (e.g., early engagement). Therefore, this finding may suggest a high level of technical stability in the execution of static phases among female players. Conversely, and from a safety perspective, the women’s cohort showed a higher incidence of red cards (0.12 vs. 0.03; *p* = 0.020). Increased disciplinary sanctions may elevate injury risk to opponents [12]. Specifically, red card offences are commonly associated with direct contact situations, exponentially increasing the probability of traumatic injury [15]. Although the absolute frequency of red cards in the analysed sample was low, this trend may reflect either stricter application of contact-related sanction frameworks in the women’s game. Scientific literature evaluating injuries and sanctions in women’s rugby [26,27] suggests that tackle height is a critical factor to consider. Female players frequently exhibit a more upright body position during the initial phase of the tackle, which significantly increases the probability of accidental head-to-head contact—a primary trigger for red cards under current high-tackle frameworks. This technical nuance highlights a specific area for coaching intervention, where emphasizing a lower body position before contact and adjusting the target zone could mitigate the risk of high-level sanctions and improve player safety.

The results of the multivariate analysis supported the univariate findings by confirming the game categories and variables contributing to both similarities and differentiation between sexes. In this regard, Total Tackles and Total Rucks showed negligible discriminatory power (r < 0.15), statistically reinforcing the premise that open-play intensity is comparable between sexes. In contrast, the distinguishing features of women’s rugby were characterised by a higher frequency of scrums (r = 0.61) and a lower prevalence of slow rucks (>6 s; r = −0.72). These findings suggest that elite women’s rugby should not be considered less intense than the men’s game, but rather different, with a distinct and well-defined structural profile, carrying direct implications for the contact exposure profile. The use of Linear Discriminant Analysis was essential to transcend variable-by-variable analysis and understand the combined structure of the game. From a practical standpoint, this analysis offers strength and conditioning coaches and technical directors a hierarchical view of which indicators carry the most weight in defining the performance profile. By identifying that higher scrum density and lower prevalence of slow rucks are the primary discriminant factors of women’s rugby, coaching staffs can design more specific and efficient training tasks, ensuring that physical and tactical preparation is aligned with the structural nature of the women’s game. To the authors’ knowledge, no previous studies have directly analysed and compared the nature of contact in elite men’s and women’s rugby union.

To the authors’ knowledge, no previous studies have directly analysed and compared the nature of contact in elite men’s and women’s rugby union. Therefore, the present study offers a novel contribution to the literature. This is further strengthened by the robustness of the sample size (*n* = 135 international matches), the use of official tracking data [18], manually validated, and the direct comparison conducted under identical laws of the game.

However, the findings should be interpreted in light of certain limitations. First, the analysis was restricted to the Six Nations Championship; therefore, extrapolation to Southern Hemisphere competitions or other domestic leagues should be undertaken with caution. Second, although the volume of contact events was quantified, the absence of integrated accelerometry or GPS data prevented the assessment of impact force magnitude (g-forces) during each collision. In future studies, the combination of precise event-based counting with sex-specific intensity metrics may allow for a more accurate characterisation of the true physiological and mechanical load experienced by players. Third, the present study employed a combination of univariate and multivariate analyses to provide a comprehensive view of gender differences, allowing for both the quantification of individual variables and the identification of distinctive game patterns. However, it must be acknowledged as a technical limitation that the analysis did not account for repeated measures or team-based clustering structures. Since the study included matches from the same nations across several editions, there is a potential interdependence in the data that could influence residual variance, an aspect that should be addressed in future research using linear mixed models to further explore the internal relationships within the sample.

While position-specific analysis provides valuable insights, the present study intentionally focused on global match demands to establish a foundational baseline for sex-based comparisons in elite rugby union. This approach was prioritized to maintain high statistical power and ensure a robust characterization of the overall game structure and disciplinary profile, particularly for infrequent but clinically relevant events like red cards. Establishing these global differences is a necessary prerequisite for future research aimed at exploring position-specific risk profiles.

## 5. Conclusions

The findings of the present study support the proposed hypothesis, demonstrating that elite women’s rugby union presents a contact-demand profile that differs structurally from the male game model, not in Open-Play variables, but in specific indicators within the Static Phases and Discipline categories. Specifically, our multivariate analysis confirms that while both sexes share a similar collision volume in open play, the female profile is uniquely defined by higher intensity in ruck continuity and greater static loading. These structural distinctions, primarily driven by scrum frequency and ruck tempo, provide the statistical evidence for a sex-specific contact profile. Accordingly, female players are exposed to a volume of tackles and rucks comparable to that of men, while simultaneously managing a significantly higher load in set-pieces, specifically characterized by a higher frequency of scrums and a faster ruck tempo. Furthermore, the results reveal a distinct disciplinary profile in the women’s game, marked by a higher incidence of red cards alongside a lower frequency of technical free kicks conceded. These findings refute the assumption of lower contact intensity in women’s rugby and demonstrate the existence of a distinct performance game profile that must be specifically analysed to appropriately tailor prevention strategies based on contact exposure and ensure safe return-to-play processes based on the demands of the women’s game, rather than extrapolated from male-derived data.

## 6. Practical Applications

The findings of this study allow for the establishment of specific guidelines to optimize physical preparation and safety in elite women’s rugby. First, given the significantly higher frequency of scrums compared to the male model, it is essential to implement specialized strength programs for forwards that prioritize isometric neck muscle resistance and trunk stability to better tolerate repeated axial loading and mitigate injury risk. Additionally, in light of the higher incidence of red cards observed in the female cohort, technical coaching should focus on adjusting the “contact zone” by retraining players to adopt a lower body position during the tackle, thereby reducing the risk of accidental head impacts and improving overall safety. Finally, coaching staffs must differentiate post-match recovery strategies; while the male game model is often driven by territorial management, the female profile is more dependent on the neuromuscular load derived from frequent static phases and a high ruck tempo, necessitating recovery protocols tailored to these specific structural demands.

## Figures and Tables

**Table 1:** Operational definitions, categorization, and abbreviations of performance variables related to physical contact and injury risk.

Categories	Variable	Definition
Open-Play	Total Tackles (TT)	The sum of all tackle attempts recorded. A tackle occurs when the ball carrier is held by one or more opponents and is brought to ground (World Rugby Law 14 *).
	Tackles Made (TM)	Number of tackles where the ball carrier was successfully stopped or brought to ground.
	Tackles Missed (TMiss)	Number of instances where a defender attempted to tackle but failed to stop the ball carrier’s progress.
	Tackle Success (TS)	Percentage calculated as (TacklesMade/TotalTackles) × 100.
	Total Rucks (RT)	Aggregate frequency of ruck phases formed. A ruck is formed when at least one player from each team are on their feet, in physical contact, close around the ball on the ground (World Rugby Law 15 *).
	Rucks Won (RW)	Rucks where the team in possession retains control of the ball.
	Rucks Lost (RL)	Rucks where possession is turned over to the opposition.
	Breakdown Steals (BS)	Number of times possession was won by the defending team directly during a ruck phase (e.g., “jackal”).
	Ruck 0–3 s (R0–3)	Percentage of rucks where the ball is available or cleared within 0 to 3 s.
	Ruck 4–6 s (R 4–6)	Percentage of rucks where the ball is available or cleared within 4 to 6 s.
	Ruck > 6 s (R > 6)	Percentage of rucks where the ball is available or cleared after more than 6 s.
Static Phases	Total Scrums (ST)	Frequency of scrum set-pieces awarded awarded/ordered to restart play after a minor infringement (World Rugby Law 19 *). Each awarded scrum is counted as a single event, excluding subsequent resets within the same sequence
	Scrums Won (SW)	Scrums where the team putting in the ball retains possession.
	Scrums Lost (SL)	Scrums where the team putting in the ball loses possession against the head.
	Total Mauls (MT)	Frequency of maul formations. A maul begins when a ball carrier is held by one or more opponents, and one or more of the ball carrier’s teammates bind on (World Rugby Law 16 *).
	Mauls Won (MW)	Mauls where the team in possession retains control or advances successfully.
	Mauls Lost (ML)	Mauls where possession is turned over to the opposition.
Discipline	Penalties Conceded (PC)	Sanctions awarded against a team for major infringements (World Rugby Law 20 *).
	Offensive Penalties (PO)	Penalties conceded by the team in possession of the ball.
	Defensive Penalties (PD)	Penalties conceded by the team not in possession of the ball.
	Free Kicks Conceded (FKC)	Sanctions awarded for technical infringements (e.g., early engagement) (World Rugby Law 20 *).
	Yellow Cards (YC)	Temporary suspension of a player for 10 min due to Foul Play (World Rugby Law 9 *).
	Red Cards (RD)	Permanent exclusion of a player from the match due to severe Foul Play (World Rugby Law 9 *).

* Definitions adapted from World Rugby Laws of the Game (2024).

**Table 2:** Comparative Analysis of Match Performance Indicators Between Elite Male and Female Rugby Union Players.

Variable	Men (Mean, SD)	Women (Mean, SD)	*p*-Value	Effect Size
Open-play				
Total Tackles (Attempts)	163.73 ± 41.28	168.26 ± 42.58	0.378	−0.11
Tackles Made (*n*)	142.29 ± 36.86	146.76 ± 37.78	0.328	−0.12
Tackles Missed (*n*)	21.45 ± 8.40	21.50 ± 10.90	0.505	−0.05
Tackle Success (%)	69.59 ± 34.67	66.80 ± 36.81	0.523	0.08
Total Rucks (*n*)	91.95 ± 22.62	90.53 ± 26.12	0.634	0.06
Rucks Won (*n*)	88.28 ± 22.38	86.91 ± 25.50	0.639	0.06
Rucks Lost (*n*)	3.67 ± 1.92	3.62 ± 2.05	0.771	−0.02
Breakdown Steals (*n*)	3.05 ± 1.64	3.41 ± 2.03	0.158	0.10
Ruck 0–3 s (%)	46.13 ± 24.66	46.12 ± 26.45	0.869	0.01
Ruck 4–6 s (%)	21.69 ± 12.35	25.88 ± 16.02	<0.001	0.25
Ruck > 6 s (%)	12.38 ± 8.98	6.11 ± 4.91	<0.001	−0.46
Static phases				
Total Mauls (*n*)	5.31 ± 2.77	5.17 ± 3.00	0.572	−0.04
Mauls Won (*n*)	4.85 ± 2.64	4.62 ± 2.80	0.413	−0.06
Mauls Lost (*n*)	0.47 ± 0.70	0.54 ± 1.15	0.976	−0.00
Total Scrums (*n*)	5.38 ± 2.51	7.21 ± 2.61	<0.001	0.43
Scrums Won (*n*)	4.95 ± 2.33	6.34 ± 2.74	<0.001	0.34
Scrums Lost (*n*)	0.43 ± 0.68	0.87 ± 1.86	0.148	0.09
Discipline				
Total Penalties Conceded (*n*)	10.05 ± 3.71	10.67 ± 3.29	0.238	0.08
Offensive Penalties (*n*)	3.53 ± 1.98	3.83 ± 1.98	0.258	0.08
Defensive Penalties (*n*)	6.65 ± 2.88	6.69 ± 2.79	0.627	0.03
Free Kicks Conceded (*n*)	0.87 ± 1.70	0.40 ± 0.63	0.003	−0.19
Yellow Cards (*n*)	0.41 ± 0.97	0.53 ± 1.48	0.749	0.02
Red Cards (*n*)	0.03 ± 0.21	0.12 ± 0.42	0.020	0.07

* Values are presented as Mean (SD). *p*-values in bold indicate statistical significance (*p* < 0.05). Effect sizes represent Cohen’s d or rank-biserial correlation depending on the statistical test applied. *n* = count/number of events.

## Data Availability

The data presented in this study are available from the corresponding author upon reasonable request. And the dataset has a Handle link: http://hdl.handle.net/10201/155010, accessed on 18 May 2026.

## Supplementary Materials

The following supporting information can be downloaded at: https://www.mdpi.com/article/10.3390/sports14050210/s1, Table S1: STROBE Statement—Checklist of items that should be included in reports of cross-sectional studies. Table S2: SORT Statement—Checklist.

## Abbreviations

The following abbreviations are used in this manuscript:

ACL	Anterior cruciate ligament
BS	Breakdown Steals
ES	Effect Size
FKS	Free Kicks Conceded
ICC	Intraclass Correlation Coefficient
LDA	Linear Discriminant Analysis
ML	Mauls Lost
MT	Total Mauls
MW	Mauls Won
PC	Penalties Conceded
PD	Defensive Penalties
PO	Offensive Penalties
RL	Rucks Lost
RT	Total Rucks
RW	Rucks Won
R0–3	Ruck 0–3 s
R4–6	Ruck 4–6 s
R > 6	Ruck >6 s
SD	Standard Deviation
SL	Scrums Lost
ST	Total Scrums
SW	Scrums Won
TM	Tackles Made
TMiss	Tackles Missed
TS	Tackle Success
TT	Total Tackles
YC	Yellow Cards
RD	Red Cards
