# Supplementary material for: Collision Volume and Contact Exposure Profile in Elite Women’s Rugby Union: Differences Compared with Men
Source: Sports (Basel). 2026 May 19;14(5):210. doi: 10.3390/sports14050210 (PMC13210970; doi:10.3390/sports14050210)
Supplement: Supplementary file 1 [file sports-14-00210-s001.zip › sports-4284220-Table S2.pdf]

The findings of this study provide a **Strength of Recommendation (SOR) Grade C** for the development of sex-specific training and injury prevention protocols in elite rugby union. Although the evidence is classified as **Level 3** due to its observational and retrospective nature, the results demonstrate consistent differences in collision demands—specifically in **Static Loading** (scrums) and **Open-Play** (rucks/mauls)—between male and female players.

From a clinical perspective, these data represent **Disease-Oriented Evidence (DOE)**, as they identify specific mechanical and frequency-based exposure risks that are precursors to injury. Clinicians and strength and conditioning coaches should consider these findings to implement targeted cervical strengthening and technical preparation for female forwards, who face unique static-pushing demands despite lower overall match collision volumes compared to their male counterparts. Further prospective research (Level 2 evidence) is required to translate these findings into **Patient-Oriented Evidence (POEM)** by demonstrating a direct reduction in concussion rates or cervical pathologies through these sex-specific interventions.

- **Evidence Level 3**: Current findings are based on a retrospective observational cohort study comparing elite male and female collision profiles.

- **Strength of Recommendation (SOR) Grade C**: Recommendations for sex-specific training are supported by consistent observational data and expert consensus within the emerging field of women's sports science.

- **Disease-Oriented Evidence (DOE)**: The study identifies critical differences in exposure and mechanical loading (precursors to injury) rather than long-term clinical outcomes (POEM).

- **Clinical Application**: Training for female forwards should prioritize specific preparation for static loading (scrums and mauls) given the unique duration-intensity profiles identified.

Key Finding	Category	Evidence Level	SOR	Clinical Translation
Lower collision frequency in women's elite rugby across all phases of match play.	Open-Play	3	C	Adjusting training loads to reflect real match demands for female players.
Static pushing phases (scrums) show distinct duration and intensity profiles in women.	Static Phases	3	C	Need for sex-specific cervical and isometric neck conditioning.
Inaccurate load estimation when using male-derived	Methodology	3	C	Implementation of sex-specific contact monitoring systems.

metrics for female collision demands.				
