# Supplementary material for: Collision Volume and Contact Exposure Profile in Elite Women’s Rugby Union: Differences Compared with Men
Source: Sports (Basel). 2026 May 19;14(5):210. doi: 10.3390/sports14050210 (PMC13210970; doi:10.3390/sports14050210)
Supplement: Supplementary file 1 [file sports-14-00210-s001.zip › sports-4284220-Table S1.pdf]

Table S1: STROBE Statement—Checklist of items that should be included in reports of ***cross-sectional studies***

	Item No	Recommendation
**Title and abstract**	1	**Page 1 (Abstract)**. Stated as: "Study Design: Observational, retrospective, comparative cohort study"
		**Page 1 (Abstract)**. Includes Background, Methods, Results, and Conclusions
**Introduction**		
Background/rationale	2	**Page 2 (Introduction)**. Paragraphs 1-3 discuss gender differences and injury risk
Objectives	3	**Page 2 (Introduction)**. "The aim of the present study was to compare..." and "The researchers hypothesized..."
**Methods**		
Study design	4	**Page 2 (Methods - Study Design)**. "Observational, retrospective, and cross-sectional design"
Setting	5	**Page 2 (Methods - Participants and Sample)**. "Six Nations Championship... 2021–2025 editions"
Participants	6	**Page 2 (Methods - Participants and Sample)**. "135 matches involving the Men's and Women's national teams"
Variables	7	**Page 3 (Methods - Variables)**. See **Table 1** for operational definitions of Open-Play, Static Phases, and Discipline.
Data sources/ measurement	8*	**Page 2 (Methods - Data Collection)**. Data collected using the Hawk-Eye tracking system.
Bias	9	**Page 2 (Methods - Data Collection)**. Concurrent manual verification was conducted on a subsample to assess reliability (ICC)
Study size	10	**Page 2 (Methods - Participants and Sample)**. Total sample of 270 team-game observations from 135 matches.
Quantitative variables	11	**Page 3-4 (Methods - Variables / Statistical Analysis)**. Variables grouped into categories (Open-Play, Static Phases, Discipline)
Statistical methods	12	(*a*) **Page 4 (Methods - Statistical Analysis)**. Shapiro-Wilk, T-tests/Mann-Whitney U, Cohen's d, and Linear Discriminant Analysis (LDA) .
		(*b*) **Page 4 (Methods - Statistical Analysis)** The study design is based on a direct comparison between two subgroups: Men's and Women's national teams. Statistical comparisons (t-tests/Mann-Whitney U) and Linear Discriminant Analysis (LDA) were specifically used to identify differences and interactions between variables characterizing these two groups.
		(*c*) **Page 4 (Methods - Statistical Analysis)** Not explicitly stated. The study utilizes official match tracking data (Hawk-Eye) for all 135 matches, implying a complete dataset for the selected metrics.
		(*d*) **Page 4 (Methods - Statistical Analysis)** The study analyzed all matches from the specified tournaments (census of the 2021-2025 Six Nations), analyzing them as 270 team-game observations. No complex survey weighting was required.
		(*e*) **Page 4 (Methods - Statistical Analysis)** To ensure data quality before the main analysis, a sensitivity/reliability check was performed on a subsample of 30 matches (22% of total) comparing the automated Hawk-Eye data against manual coding by an expert, yielding ICC values between .78 and .86.
**Results**		
Participants	13*	(a) **Page 2 (Methods - Participants and Sample)** The study included a total sample

		of 270 team-game observations derived from 135 international matches involving the Men's and Women's national teams participating in the 2021–2025 editions of the Six Nations Championship. All 270 observations were included in the final analysis.
		(b) **Page 2 (Participants and Sample / Data Collection): Paragraphs 1-2:** No non-participation or data loss was reported. Since the study followed a retrospective design using official performance data (Hawk-Eye system) from a professional competition, there were no player dropouts or exclusions of eligible matches.
		(c) A flow diagram was not included in the manuscript. Given the straightforward nature of the sample selection (census of matches within specific tournament years without exclusion criteria), a diagram was likely deemed unnecessary.
Descriptive data	14*	(a) **Page 4 (Results - Descriptive and Univariate Analysis):** Table 2: The study provides descriptive characteristics of match performance indicators categorized by sex (Men vs. Women). **Table 2** presents the Mean ± Standard Deviation (SD) for all key variables across three categories: Open-Play (e.g., Total Tackles, Rucks), Static Phases (e.g., Scrums, Mauls), and Discipline (e.g., Penalties, Cards).
		(b) **Page 4 (Results):** No missing data were reported for the variables of interest. The analysis was conducted on a complete dataset of 270 team-game observations derived from official tracking data (Hawk-Eye), implying full data availability for all analyzed metrics
Outcome data	15*	**Page 4 (Results)**. See **Table 2** for counts of Tackles, Rucks, Scrums, etc.
Main results	16	(*a*) **Page 4 (Table 2).** The study reports unadjusted estimates for all performance variables. Table 2 presents the Mean ± Standard Deviation (SD) for Men and Women. Precision and significance are reported using P-values and Effect Sizes (Cohen's d or rank-biserial correlation) rather than 95% Confidence Intervals
		(*b*) **Page 3 (Table 1).** Continuous time variables for Ruck duration were categorized into three specific time bands for analysis: "Fast Rucks" (0–3 seconds), "Medium Rucks" (4–6 seconds), and "Slow Rucks" (>6 seconds)
		(*c*) Not applicable. This study analyzes performance indicators (frequencies of game events) rather than epidemiological risk ratios or odds ratios for disease/injury outcomes.
Other analyses	17	**Page 5 (Results - Multivariate Analysis)**. Linear Discriminant Analysis (LDA) and Figure 2 .
**Discussion**		
Key results	18	**Page 5 (Discussion)**. Paragraph 1 summarizes findings refuting the initial hypothesis.
Limitations	19	**Page 6 (Discussion - Limitations)**. Mentions geographic restriction and lack of g-force data.
Interpretation	20	**Page 5-6 (Discussion)**. Interpretation of contact load and structural differences between sexes.
Generalisability	21	**Page 6 (Conclusions)**. Findings apply to "Elite women's rugby"
**Other information**		
Funding	22	**Not Applicable**. No funding section found in the provided text, only Data Availability.

*Give information separately for exposed and unexposed groups.

Note: An Explanation and Elaboration article discusses each checklist item and gives methodological background and published examples of transparent reporting. The STROBE checklist is best used in conjunction with this article (freely

available on the Web sites of PLoS Medicine at http://www.plosmedicine.org/, Annals of Internal Medicine at http://www.annals.org/, and Epidemiology at http://www.epidem.com/). Information on the STROBE Initiative is available at www.strobe-statement.org.
